# Endoscopic submucosal dissection combined with mini-probe endoscopic ultrasonography to remove a fishbone in the muscularis propria

**DOI:** 10.1055/a-2436-1482

**Published:** 2024-10-25

**Authors:** Bin-Yang Luo, Zhu Wang

**Affiliations:** 1 Department of Gastroenterology and Hepatology, West China Hospital, Sichuan University, Chengdu, China

Fishbones are a common gastric foreign body. Once they penetrate the gastric wall, endoscopic removal with forceps often fails. If the foreign body is located within the submucosa, dissecting along the submucosal layer aids in lesion identification and removal 1 2 . However, when the foreign body has penetrated more deeply into the muscularis propria or beyond, a linear endoscopic ultrasound (EUS) or laparoscopic approach may be required 3 4 . We present a method combining endoscopic submucosal dissection (ESD) with mini-probe EUS for deep foreign body retrieval.

A 71-year-old woman with a 2-month history of abdominal discomfort underwent computed tomography imaging, which revealed a 3-cm high-density foreign body penetrating the gastric antrum and extending into the pancreatic head. Gastroscopy identified an ulcer on the lesser curvature of the antrum, suspected of being the puncture site. EUS localized the foreign body embedded in the muscularis propria adjacent to the ulcer in the anterior wall ( Fig. 1 ).

**Fig. 1:**
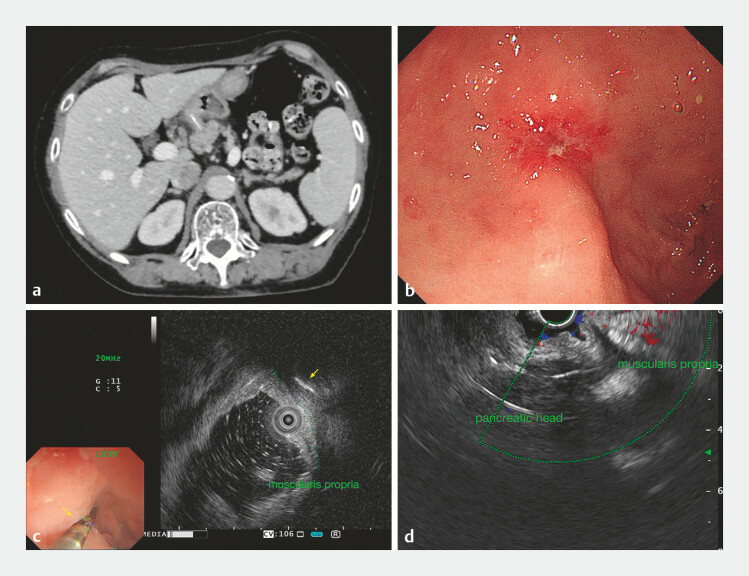
Imaging and investigations. **a** Enhanced computed tomography scan revealed a 3-cm high-density strip penetrating the gastric antrum and extending into the pancreatic head. **b** Gastroscopy found a small ulcer at the lesser curvature of the antrum. **c, d** Endoscopic ultrasound revealed the foreign body (yellow arrow) embedded in the muscularis layer near the ulcer and penetrating the pancreas.

Submucosal injection and incision with a HookKnife (KD-620LR; Olympus, Tokyo, Japan) allowed for submucosal exploration, during which the adherent area around the ulcer was incised under clip traction. The muscularis propria was exposed but the foreign body was not immediately visible. Re-localization with the mini-probe guided a targeted incision, enabling complete removal of the foreign body ( Fig. 2 ). The injured muscle layer was closed with clips ( Video 1 ). The patient was administrated omeprazole and cefoxitin. She was successfully discharged on the third postoperative day.

**Fig. 2:**
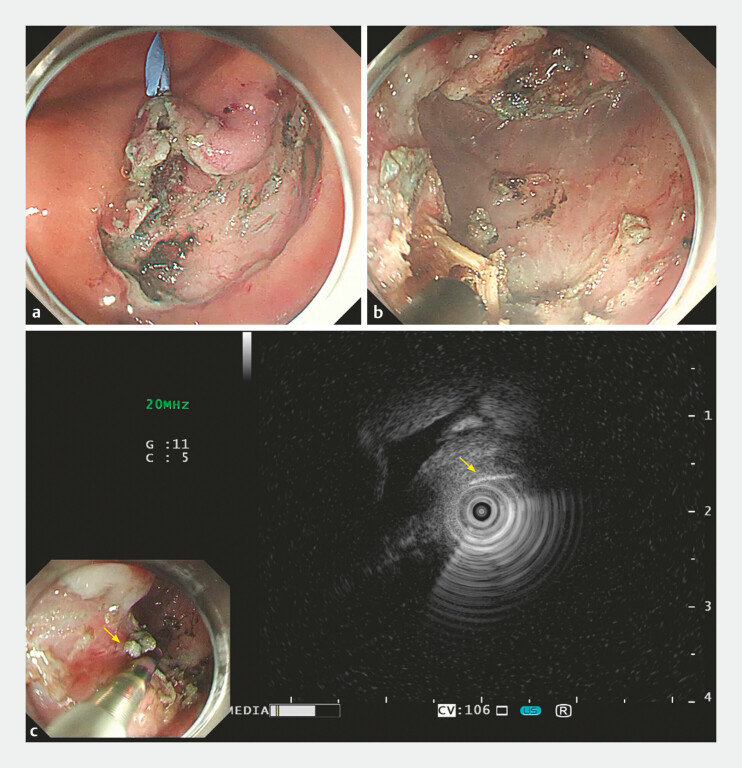
**a** Endoscopic submucosal dissection exposed the muscularis layer, but the foreign body was not visible. **b, c** Following careful incision under ultrasound guidance, the foreign body was successfully retrieved.

Endoscopic submucosal dissection combined with mini-probe endoscopic ultrasound to remove a fishbone hidden in the muscularis propria.Video 1

The primary challenge in removing a foreign body embedded in the gastric wall lies in accurately locating the lesion and determining the precise incision site. When the foreign body is situated in the submucosa, ESD can create a submucosal tunnel to locate the fishbone. However, for cases involving deeper penetration, the use of mini-probe EUS aids in pinpointing the incision site, thereby avoiding unnecessary full-thickness resection and minimizing secondary injury.

Endoscopy_UCTN_Code_TTT_1AO_2AG_3AD

## Notes

Endoscopy E-Videos https://eref.thieme.de/e-videos
*E-Videos* is an open access online section of the journal *Endoscopy* , reporting on interesting cases and new techniques in gastroenterological endoscopy. All papers include a high-quality video and are published with a Creative Commons CC-BY license. Endoscopy E-Videos qualify for HINARI discounts and waivers and eligibility is automatically checked during the submission process. We grant 100% waivers to articles whose corresponding authors are based in Group A countries and 50% waivers to those who are based in Group B countries as classified by Research4Life (see: https://www.research4life.org/access/eligibility/ ). This section has its own submission website at https://mc.manuscriptcentral.com/e-videos .
